# What do older adults with multimorbidity and polypharmacy think about deprescribing? The LESS study - a primary care-based survey

**DOI:** 10.1186/s12877-020-01843-x

**Published:** 2020-10-31

**Authors:** Zsofia Rozsnyai, Katharina Tabea Jungo, Emily Reeve, Rosalinde K. E. Poortvliet, Nicolas Rodondi, Jacobijn Gussekloo, Sven Streit

**Affiliations:** 1 Institute of Primary Health Care Bern (BIHAM), University of Bern, Mittelstrasse 43, 3012 Bern, Switzerland; 2 Graduate School for Health Sciences, University of Bern, Bern, Switzerland; 3 Quality Use of Medicines and Pharmacy Research Centre, UniSA: Clinical and Health Sciences, University of South Australia, Adelaide, SA Australia; 4 NHMRC Cognitive Decline Partnership Centre, Kolling Institute of Medical Research, Northern Clinical School, Faculty of Medicine and Health, University of Sydney, Sydney, Australia; 5 Geriatric Medicine Research and College of Pharmacy, Dalhousie University and Nova Scotia Health Authority, Halifax, NS Canada; 6 Department of Public Health and Primary Care, Leiden University Medical Center, Leiden, The Netherlands; 7 Department of General Internal Medicine, Inselspital, Bern University Hospital, University of Bern, Bern, Switzerland; 8 Department of Internal Medicine, Section Gerontology and Geriatrics, Leiden University Medical Center, Leiden, The Netherlands

**Keywords:** Deprescribing, Polypharmacy, Multimorbidity, Patient attitudes, Older adults, General practice

## Abstract

**Background:**

Multimorbidity and polypharmacy are very common in older adults in primary care. Ideally, general practitioners (GPs), should regularly review medication lists to identify inappropriate medication(s) and, where appropriate, deprescribe. However, it remains challenging to deprescribe given time constraints and few recommendations from guidelines. Further, patient related barriers and enablers to deprescribing have to be accounted for. The aim of this study was to identify barriers and enablers to deprescribing as reported by older adults with polypharmacy and multimorbidity.

**Methods:**

We conducted a survey among participants aged ≥70 years, with multimorbidity (≥3 chronic conditions) and polypharmacy (≥5 chronic medications). We invited Swiss GPs, to recruit eligible patients who then completed a paper-based survey on demographics, medications and chronic conditions. We used the revised Patients’ Attitudes Towards Deprescribing (rPATD) questionnaire and added twelve additional Likert scale questions and two open-ended questions to assess barriers and enablers towards deprescribing, which we coded and categorized into meaningful themes.

**Result:**

Sixty four Swiss GPs consented to recruit 5–6 patients each and returned 300 participant responses. Participants were 79.1 years (SD 5.7), 47% female, 34% lived alone, and 86% managed their medications themselves. Sixty-seven percent of participants took 5–9 regular medicines and 24% took ≥10 medicines. The majority of participants (77%) were willing to deprescribe one or more of their medicines if their doctor said it was possible. There was no association with sex, age or the number of medicines and willingness to deprescribe. After adjustment for baseline characteristics, there was a strong positive association between willingness to deprescribe and saying that because they have a good relationship with their GP, they would feel that deprescribing was safe OR 11.3 (95% CI: 4.64–27.3) and agreeing that they would be willing to deprescribe if new studies showed an avoidable risk OR 8.0 (95% CI 3.79–16.9). From the open questions, the most mentioned barriers towards deprescribing were patients feeling well on their current medicines and being convinced that they need all their medicines.

**Conclusions:**

Most older adults with polypharmacy are willing to deprescribe. GPs may be able to increase deprescribing by building trust with their patients and communicating evidence about the risks of medication use.

**Supplementary Information:**

The online version contains supplementary material available at 10.1186/s12877-020-01843-x.

## Background

Managing patients with multimorbidity (≥3 chronic conditions) has become the norm for general practitioners (GPs). In the UK for example, three quarters of consultations involve patients with multimorbidity [1]. Multimorbidity is strongly associated with age and with polypharmacy (often defined as ≥5 chronic medicines [2]). Currently, treatment guidelines are mainly based on the management of single diseases and on evidence from trials that often exclude older patients with multimorbidity [3] Therefore, recommendations for individual medical conditions often fail to consider competing factors, such as drug-disease interactions and risks due to polypharmacy [4]. As a result, the prevalence of polypharmacy is on the rise, especially in patients with multimorbidity. With this comes an increased risk for potentially inappropriate medications (PIMs); these are medications where the potential risk outweighs the potential benefit in the individual. The possible consequences of polypharmacy and PIMs use include increased risk of adverse drug events [5], medicine errors [6], adverse drug reactions [7], poor adherence [8], and impaired quality of life [9], especially in older multimorbid patients [10].

Deprescribing is the withdrawal of PIMs with the goal of managing polypharmacy and improving outcomes [11]. Deprescribing is relevant and should be considered for all patients who may be taking an inappropriate medicine [12]. However, certain medications are often targeted, such as high risk medicines in frail older adults, preventive medicines in patients with limited life expectancy [13] including people with cancer under palliative care [14, 15].

While the rationale to deprescribe is clear, the implementation is less clear and in practice, important barriers exist for physicians and patients [16]. Physicians have reported that patient resistance or unwillingness to deprescribe is a major barrier to deprescribing in practice [17]. Studies with patients have found that [18] fear, lack of knowledge on how to deprescribe and belief that their medicines are appropriate are barriers to their willingness to have a medication deprescribed. Despite these barriers, older adults have reported willingness towards deprescribing when their health care professional is supportive [19]. Barriers and enablers for deprescribing might differ by countries, cultures and local health care systems. Patient willingness to deprescribe has been previously studied in several countries, however, not all participants had polypharmacy and multimorbidity and were often not recruited from primary care [20–22] leaving a gap in understanding the perspective of older patients with multimorbidity and polypharmacy in general practice.

This study aims to determine the willingness towards deprescribing in older adults with polypharmacy in Switzerland, who are at high risk for potentially inappropriate medication and would therefore likely benefit from deprescribing. We further wanted to learn which potential barriers and enablers are most prevalent in this population and to explore their association with the reported willingness to deprescribe.

## Methods

### Design

Cross-sectional survey among patients in general practice using anonymous paper based questionnaires distributed by their GPs from May 2018 to February 2019.

### Study population and processes

Inclusion criteria for patients were age 70 years or older, multimorbidity (three or more chronic conditions), and polypharmacy (regular intake of five or more chronic medicines). Furthermore, patients had to be able to read and write in German language. There were no exclusion criteria.

Patients were recruited by GPs. We invited Swiss GPs to each recruit five to six eligible patients. Switzerland has no registry of GPs which made it impossible to select a random sample. We therefore asked GPs who participated in a former study using an online questionnaire on attitudes of GPs towards deprescribing [23] if they would assist in recruitment of patients. We also allowed other GPs interested to participate through advertising the study at Primary Care institutes and in GP quality circles. All participating practices were paid 100 Swiss Francs (about 100 Euros) to compensate for time required for screening, obtaining informed consent and other recruitment related activities. The Ethics committee of the Canton of Bern approved the study. (Nr: 2017–02188).

Participating GPs were instructed to *consecutively* screen for eligible patients during their regular consultation program to limit selection bias. GPs recorded the number of screened patients, number of eligible patients and number of those not willing to participate.

All patients gave written informed consent to participate before receiving the paper-based study questionnaire. Patients were invited to answer the questionnaire in the waiting room or at home anonymously and return the survey to the medical assistant of their practices (who then returned them as a batch to the study team).

### Questionnaire

- We used the revised Patients’ Attitudes Towards Deprescribing (rPATD) questionnaire for older adults which contains twenty-two 5-point Likert scale questions on attitudes and beliefs about their medications and deprescribing. The rPATD was developed and validated in Australia [24] and has been employed in various countries and settings [20, 21, 24]. The rPATD contains four factors with five questions per factor (involvement, burden, appropriateness, and concerns about stopping) as well as two global questions. We translated the original questions from the rPATD from English into German. This then underwent independent back-translation from German to English by an individual who had not seen the original English version. The translation and back-translations were then reviewed by the research team (including the primary author of the original English rPATD, ER) with discussion and editing of the German version to resolve any concerns about the translation. As one of the aims of the study was to quantify the barriers to and enablers of deprescribing, 12 questions were added to the questionnaire to cover topics important to patients in a primary care setting identified in previous qualitative research [18]. These additional questions were developed by members of the research team (SS, NR, RKEP) and chosen for inclusion in the study due to their perceived relevance to the local context of primary care in the German speaking part of Switzerland. The questions were not chosen according to themes that were not represented in the rPATD (as the factors of the rPATD are closely aligned with the themes from this systematic review), and instead were to broaden capturing of attitudes within the themes. Where possible, the wording of the question was kept as close to the quotes from older adults in the original research studies included in the systematic review, however, wording was developed and refined by the research team. The possible answers for all questions were: strongly agree - agree – unsure – disagree – strongly disagree.
- Two additional open-ended questions on other barriers and enablers towards the willingness to deprescribing were also added to capture potential barriers and enablers not included in the rPATD or additional quantitative questions (“Do you think there are other reasons why you wouldn’t reduce or stop medicines?” and “Do you think there are other reasons why you would like to reduce or stop medicines?”). We then piloted the translated questionnaire and additional questions with four eligible patients for comprehensibility (no changes were required).

We also collected self-reported data:

- demographic data: age, sex, living status and involvement in medication management.
- full list of current chronic medicines (intake of > 6 months). If participants had trouble self-completing this list, they could seek help from their GP
- chronic conditions. We provided participants a list of most prevalent chronic conditions in patient-friendly language [25]. Participants could tick boxes, if they were diagnosed with those diseases.

We used the STROBE statement checklist to report our study findings [26].

Preliminary results of this project were presented at the annual meeting of the European General Practice Research Network in Tampere in May 2019 [27].

### Statistical analysis

Our main outcome was willingness towards deprescribing, which was measured with the question from the rPATD: *‘If my doctor said it was possible, I would be willing to stop one or more of my regular medicines’*. If participants answered, “strongly agree or agree” they were considered to be willing to deprescribe.

We used descriptive statistics to report baseline characteristics of our sample stratified by willingness to deprescribe. To compare participants who were willing to deprescribe versus not willing to deprescribe, we used t-test and Chi2 test where appropriate. Likert-scale answers from rPATD and the additional questions about deprescribing were dichotomized from the 5-point Likert scale responses into “strongly agree/agree” versus “unsure/disagree/strongly disagree” for analysis. Different members of the study team then separately categorized them as enablers, barriers or involvement-related and then discussed potential disagreement until agreement was reached. These categories were formed for the case if the question was answered with “strongly agree/agree” (e.g. “I don’t like to take medicines”, if answered with “strongly agree/agree” the statement qualifies as an enabler towards deprescribing, but if answered with “unsure/disagree/strongly disagree” it is not necessarily a barrier).

To adjust for baseline characteristics, we used a multivariable mixed-effects logistic regression model that also accounted for possible clustering within each GP as a random-effect. The same model was used to assess the associations of items from the questionnaire and willingness to deprescribe. In the model we adjusted a priori for sex and age and for baseline characteristics if there was a significant difference (*p*-value< 0.05) between those willing and those not willing to deprescribe.

The first and last author analyzed the responses to the open-ended questions. They coded and categorized answers to the open questions (*“Do you think there are other reasons why you wouldn’t reduce or stop medicines?” and “Do you think there are other reasons why you would like to reduce or stop medicines?)* into meaningful themes. Disagreements were resolved by consensus. After creating the list of themes, it was counted how often the topics were mentioned by participants. Multiple answers were possible per participant.

A *p*-value < 0.05 was considered significant. Data management was done using EpiData Manager and EpiData Entry Client v.4.4.1.0 (EpiData Association, Denmark). Statistics were computed using Stata 15.02 (StataCorp, College Station, TX, USA) for all analyses.

## Results

We invited 830 Swiss GPs from all over the German part of Switzerland to participate. Out of these, 64 (7.7%) GPs recruited participants. Reasons why GPs did not participate included lack of time, health issues or personnel shortage in their practice. Participating GPs and therefore their patients came from all over the German speaking part of Switzerland.

**Fig. 1:**
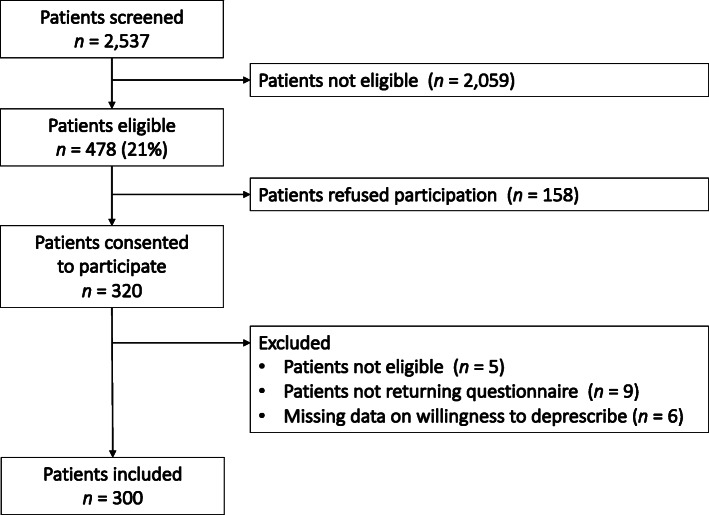
Flow chart of study participants

Target recruitment was 5 to 6 patients per GP. During the screening period, the 64 GPs screened 2537 consecutive patients for eligibility, of those 531 (21%) met the inclusion criteria. Ultimately 300 participants were included in this analysis. Reasons for non-participation and a study flow chart is provided in Fig. 1.

### Participant characteristics

Table 1 describes the baseline characteristics of our participants: 47% were female, with a mean age of 79.1 years (SD, 5.7). Thirty-four percent were living alone and 86% managed their own medications*.* Twenty-nine percent had no further or vocational education after obligatory school; 49% had done an apprenticeship (vocational education) and another 22% had higher education.

**Table 1:** Baseline characteristics of study participants stratified by willingness to deprescribe

Baseline characteristics	Overall *n* = 300	Willing to deprescribe^a^ *n* = 231 (77%)	Not willing to deprescribe^a^ *n* = 69 (23%)	*p*-value
Female, n (%) (*n* = 300)^b^	141 (47)	104 (45)	37 (54)	0.21
Age, mean (SD) (*n* = 292)	79.1 (5.7)	78.9 (5.7)	79.8 (5.8)	0.24
Living alone, n (%) (*n* = 298)	100 (34)	76 (33)	24 (35)	0.81
Self-management of medication, n (%) (n = 298)	256 (86)	196 (86)	60 (87)	0.78
Education level, n (%) (*n* = 299)				0.006
obligatory education	86 (29)	57 (25)	29 (42)	
Apprenticeship	146 (49)	114 (49)	32 (46)	
Higher education	67 (22)	59 (26)	8 (12)	
Number of medicines, mean (SD) (*n* = 294)	8.0 (2.8)	8.0 (2.7)	8.1 (2.9)	0.89
5–9 medicines	228 (76)	176 (76)	52 (75)	
≥ 10 medicines	72 (24)	52 (24)	13 (25)	0.89

*SD* standard deviation

^a^Willing to deprescribe, when answering true/rather true and not willing to deprescribe, when answering don’t know/rather not true/not true to the question: “*If my doctor said, it was possible I would be willing to stop one or more of my regular medicines’* “

^b^numbers report the number of patients with no missing information on the respective variable

Participants had a mean number of 3.3 (SD 1.3) chronic conditions from our list of common chronic conditions in ambulatory care. The mean number of medicines was 8.0 (SD 2.8) with the largest number of medicines taken by a single participant being 22 medicines. Twenty-four percent had excessive polypharmacy [28], defined as taking 10 or more regular medicines. The majority of our sample were willing to deprescribe (77%). There was no significant differences in willingness to deprescribe based on participant characteristics, except for educational level. A greater proportion of participants who were willing to deprescribe had higher education compared to those who were not willing to deprescribe (26% vs. 12%, *p* = 0.006).

### Barriers and enablers towards deprescribing

**Fig. 2:**
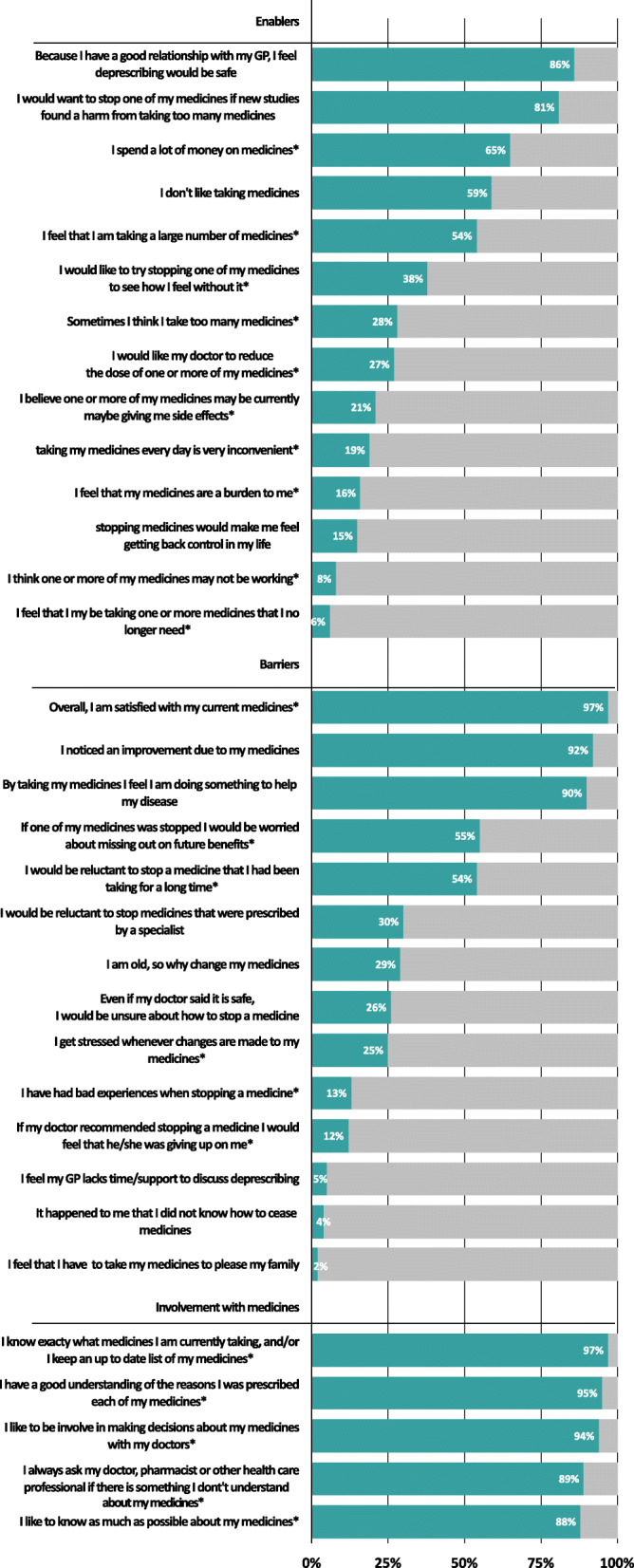
Enabler, barrier and involvement items, sorted by proportion of patients agreeing with questions per domain; Legend: Enabler, barrier and involvement items from questionnaire, agreed or strongly agreed on (coloured part of the bar) versus unsure, disagreed, strongly disagreed on (grey part of the bar) by patients with multimorbidity and polypharmacy. Items are sorted by proportion of patients agreeing with questions per domain. * from rPATD

Figure 2 describes results of the enablers, barriers and other questions related to deprescribing. Among the enablers, most participants reported that they had a good relationship with their GP and therefore felt that deprescribing was safe (86%) and agreed that if new studies found harm from taking too many medicines, they would want to deprescribe (81%). Out of the 14 questions categorized as barriers, the majority agreed to 5 of them. There was high satisfaction with current medications (97%) and most participants noticed an improvement when taking their medicines (92%). Only 13% had a previous bad experience with deprescribing. Participants showed a high involvement with their medicines: 97% wanted to know as much as possible about their medicines; 95% stated to understand the reasons why they were taking each of their medicines; 94% wanted to be involved in decision making about their medicines; 89% always asked a healthcare professional if they had a question about their medicines and 88% like to know as much as possible about their medicines *(*Fig. 2*).*

### Associations with willingness to deprescribe

In Table 2, we show the associations between baseline characteristics and willingness to deprescribe with an adjusted multilevel mixed-effects model. There was no significant association between age, sex, number of medicines, medication-self management, and living status with the willingness to deprescribe. Participants with higher education were more likely to be willing to deprescribe, compared to those with basic education (*p* = 0.006).

**Table 2:** Willingness to deprescribe, adjusted for patient characteristics and GP-clusters (*n* = 284)

Baseline characteristics	Adjusted^**1**^ OR (95%CI) of participants willingness to deprescribe	***p***-value^**1**^
**Sex**		
Female	0.94 (0.49–1.79)	0.84
Male	ref.	
**Age,** per year increase	0.97 (0.92–1.02)	0.29
**Living alone**		
Yes	1.27 (0.66–2.44)	0.47
No	ref.	
**Self-management of medication**		
Yes	0.79 (0.33–1.92)	0.61
No **Education level**	ref.	
Obligatory education	ref.	
Apprenticeship	1.63 (0.84–3.16)	0.15
Higher education	3.28 (1.26–8.55)	0.015
**Number of medicines**, per unit increase	1.01 (0.91–1.12)	0.92

1 Multivariable mixed-effects logistic regression model adjusting for all covariates in the table and for GP-cluster as a random-effect

Among our participants, 83 (28%) reported themselves that they had less than three chronic conditions, despite this being an inclusion criterion. As this likely indicates a problem with how this data was collected (self-report according to a pre-defined list), we did not use this variable for analysis as intended a priori.

**Fig. 3:**
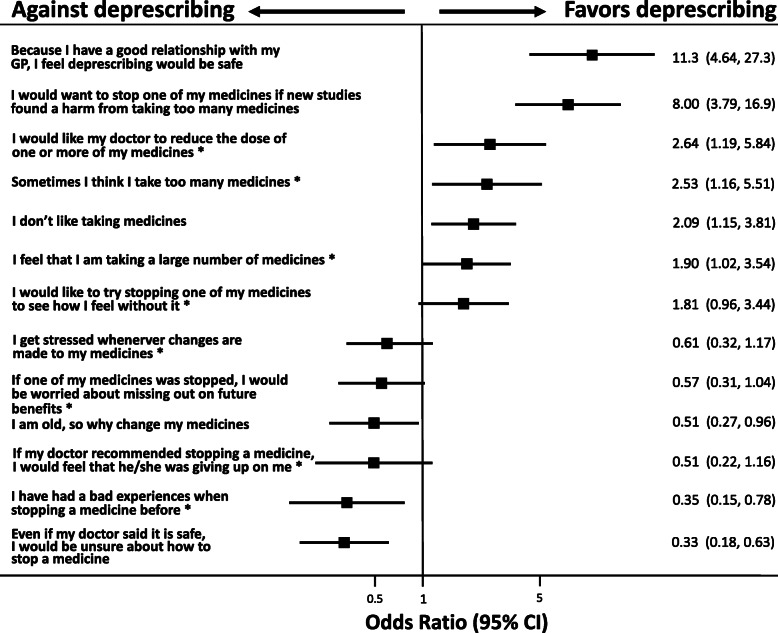
Significant enablers and barriers towards the willingness to deprescribe in a forest plot; Legend: Significant barriers and enablers towards the willingness to deprescribe. Odds ratios from a multivariable mixed-effects logistic regression model adjusted for age, sex, education level, number of medicines, living status, medication self management and GP as random-effect. OR sorted by point estimate (top-down); * from rPATD

Figure 3 shows all the questions that had a statistically significant association with willingness to deprescribe, sorted by their strength of association. There was a strong positive association with participants belief that they have a good relationship with their GP and so feel safe about deprescribing (OR 11.3, 95%CI 4.6–27.0) and that they would like to deprescribe if new studies found harm from taking too many medicines (OR 8.0, 95%CI 3.8–16.9). Other enablers were, wanting their GP to reduce the dose of their medicines (OR 2.64, 95%CI 1.2–5.8) and believing that they take too many medicines (OR 2.5, 95%CI 1.2–5.5). Important barriers with a negative association to willingness to deprescribe were being unsure about how to stop a medicine, even if their doctor said that it is safe (OR 0.33, 95%CI 0.2–0.6) and previously having had a bad experience with deprescribing (OR 0.4, 95%CI 0.2–0.8) *(*Fig. 3*).*

### Open-ended questions

The highly mentioned barriers from the open questions were participants feeling well with their current medicines and being convinced that they need all of their medicines. Other barriers were fear of recurrent symptoms or worsening of health. The most commonly mentioned enablers were experiencing a side effect or no effect from the medicines, drug-drug interactions, trust in doctors to only prescribe what is necessary and cost reduction (Table 3).

**Table 3:** Answers to open questions on other enablers and barriers towards deprescribing^a^

Enablers	Number of participants^a^
side effects, interactions or no effect of medicines, feeling bad with medicine	37
trust in doctor to deprescribe when necessary	16
cost reduction	16
**Barriers**	
feel well with current medicine, convinced that all are needed	56
fear of recurrent symptoms or worsening of health	45
trust in doctors to only prescribe what is necessary	25

^a^*179 participants replied to both open-ended questions*

^b^*More than one answer per patient possible*

## Discussion

### Summary

In a consecutive sample of Swiss primary care patients, 21% were older than 70 years and had multimorbidity and polypharmacy and were therefore eligible for inclusion in our study. In our participants, who had a mean age of 79.4 years and took an average of 8 regular medications, we found that most (77%) are willing to have a medicine deprescribed if their doctor said it was possible. Individuals with a higher level of education were more likely to be willing to deprescribe. Based on our results, the strongest enablers for patient willingness to deprescribe are having a good relationship with their GP and if new studies found harm from taking too many medicines. Barriers were previously having a bad experience with deprescribing and uncertainties about how to stop a medicine. Via our open questions, additional reported barriers were being convinced that they need all of their medicines and fear of recurrent symptoms. While noticing side effects or no effects from their medicines were reported enablers.

### Comparison to existing literature

Our most important finding, that the majority of participants were willing to deprescribe, is consistent with findings from other studies using the rPATD internationally [19, 21, 22, 29, 30]. Our population was unique in comparison to many of the earlier studies in that we only included participants with polypharmacy and multimorbidity. For example, the Australian study recruited older adults taking one or more medicines and less than half of their sample were taking 6 or more medicines [30]. Eliciting the attitudes and beliefs of older adults with polypharmacy is important because this is the population that is most likely to benefit from deprescribing and it is possible that the views of this population will differ. Older adults with polypharmacy may feel a strong dependency towards their medicines, and also might have established habits. It was interesting that in the open-ended questions a commonly reported barrier was the belief that all their medicines were necessary. While we chose to only include participants with polypharmacy, there have been inconsistent results in previous studies about the relationship between polypharmacy and patient willingness to deprescribe. In a Japanese study, a lower willingness to deprescribing in a healthier and younger population was found, with a higher willingness in older participants with more medicines [22]. Similarly, in a US study having 2 or more chronic medical conditions was found to be associated with higher willingness to deprescribe, however age was not associated [21].

Previous studies internationally have found contradictory results as to whether or not number of medicines is associated with willingness to deprescribing [19–22, 30]. We did not find an association between number of medicines and willingness to deprescribe in our population, however, as we only included participants with polypharmacy, we are not able to determine if those on less medicines had different attitudes. Willingness to deprescribe might be influenced by the individual’s perception of the number of medicines that they take. Despite all our participants having polypharmacy, only 54% felt that they took a large number of medicines. Participants who agreed that they take a large number or too many medicines were more likely to be willing to deprescribe. This is in line with a recent study in Switzerland, where patient perception of treatment burden differed significantly from the doctor’s perception. For practice, it is therefore important to find out the patient’s perceived burden, so that discussion about deprescribing can be accordingly targeted [31].

The only participant characteristic that we found to be associated with willingness to deprescribe was higher education. It may be that people who are more educated are more open to change and critical of doctors’ advice. Other baseline characteristics showed no association with willingness to deprescribe similarly to studies in Japan, Australia and USA [21, 22, 32].

The most important barriers from participant responses to our open questions were being convinced that all their medicines are necessary and fear of recurrent symptoms. This is similar to the findings of a qualitative study in Switzerland among patients who did not pursue deprescribing offers from their GP [33].

### Implications

For the future, a good knowledge of barriers and enablers from the patient view is important for developing deprescribing interventions, guidelines, and patient and clinician educational materials. Another important finding of this study was that 81% would like to deprescribe if new studies found harm from taking too many medicines. This supports the need for further research into the benefits and harms of deprescribing of different medicines and in different populations. For guidelines and patient and clinician educational materials, our results can be used to inform their content; older adults with polypharmacy and multimorbidity want to be informed about their medicines and to understand the reasons for taking them. Talking about former bad experiences and how potential negative outcomes of deprescribing will be managed is important as this was associated with reduced willingness to deprescribe. Furthermore, asking about the subjective burden of medicine intake in every patient, rather than looking at the number of medicines taken regularly, could lead to more success in deprescribing, since it was more associated with the willingness to deprescribe while number of medicines was not.

Clear instructions for participants how to cease certain medicines will also be helpful. Overall, discussing the beliefs and attitudes of patients and determining if there are any barriers towards deprescribing in the individual will enhance shared decision making and support deprescribing.

### Limitations and strengths

In this study, the screening and recruitment was done by GPs. Simple random sampling was not possible (many Swiss GPs still have paper documentation) and so we instructed the GPs to conduct consecutive sampling to identify and recruit participants to reduce selection bias. However, as this was external to the research team, we cannot completely rule out the possibility of selection bias by the GPs. Screening records from the GPs show that 21% of participants were eligible during the screening period. This number is comparable to other Swiss studies in ambulatory care on number of multimorbid participants with polypharmacy [34]. Overall, men were slightly overrepresented in our sample, but female participants were significantly older with mean age of 79.8 years (SD 6) for women, compared to Swiss census data [35]. It is also possible that patients who chose to participate may have had more favorable views about deprescribing than those who refused (33% refusal rate). Among our participants, 83 (28%) of participants reported that they had less than three chronic conditions, despite this being an inclusion criterion for screening through GPs. As this likely indicates a problem with how this data was collected (self-report according to a pre-defined list), we were not able to use this variable for analysis as intended a priori. Specifically, the under-reporting of co-morbidities is likely due to our method of checkboxes listing most prevalent chronic conditions in patient-friendly language; participants might have had conditions, which were not listed or might have known their conditions by a different name. However, this likely had little impact on our findings in regard to the results representing the attitudes of those with multimorbidity as it was the participants GP that determined their eligibility based on this criteria. Another limitation is the hypothetical and non-medicine specific nature of the rPATD and additional questions used in this study. Additionally, due to the cross-sectional nature of this study we are not able to confirm directionality (i.e. cause and effect) of the associations we identified between barriers and enablers and willingness to deprescribe. Another important limitation is that the non-rPATD additional questions did not undergo any formal validation. The two questions that were found to have the strongest associations with willingness to deprescribe (saying that because they have a good relationship with their GP, they would feel that deprescribing was safe and agreeing that they would be willing to deprescribe if new studies showed an avoidable risk) are both questions that were created just for this study (not rPATD questions). As they have not undergone validation (other than piloting in four participants), these finding should be interpreted with caution.

To our knowledge, this was the first study to use the rPATD and other questions about barriers and enablers towards deprescribing in a population most likely to have potentially inappropriate medicines in Switzerland, namely older adults with polypharmacy and multimorbidity. However, we do not know if our results are generalizable to populations outside the German speaking part of Switzerland, as we only used the German translation of the rPATD (for pragmatic reasons). By instructing GPs to recruit 5–6 eligible patients each we aimed to maximize the distribution of participants from across the German speaking part of Switzerland and prevent bias that could have been created by a small number of GPs recruiting a large proportion of the participants.

## Conclusion

Most German speaking Swiss older adults with polypharmacy are willing to deprescribe. GPs may be able to increase deprescribing by building trust with their patients and communicating evidence about the risks of medication use. Future research should explore how to best engage patients in conversations about deprescribing.

## Supplementary Information

**Additional file 1.**

## Data Availability

The dataset used and analyzed during the current study is available from the corresponding author on reasonable request.

## Abbreviations

GP: General practitioner
PIMs: Potentially inappropriate medications
rPATD: Revised patients’ attitudes towards deprescribing
