# Supplementary material for: What do older adults with multimorbidity and polypharmacy think about deprescribing? The LESS study - a primary care-based survey
Source: BMC Geriatr. 2020 Oct 31;20:435. doi: 10.1186/s12877-020-01843-x (PMC7602330; doi:10.1186/s12877-020-01843-x)
Supplement: Supplementary file 1 — Additional file 1. [file 12877_2020_1843_MOESM1_ESM.docx]

**Study About Barriers and Enablers to Deprescribing**

**Questionnaire**

*(translated from German)*

In the following, there will be a four-part questionnaire:

*Part 1, Page 2*: General questions.

*Part 2, Page 2-3*: Your current medication list.

*Part 3, Page 4-5*: Diagnoses.

*Part 4, Page 5-10*: Questions about your current medications.

We ask you to fill in all the four parts of the questionnaire and we thank you in advance for doing it.

If you have any questions about the questionnaire please contact your GP.

Please return the fulfilled questionnaire to your GP’s office.

Best regards

**Part 1. General information.**

- Your age (in years): ____________
- Sex: □ female □ male
- Place of living (ZIP code): ­­­­­­­­­­­­­­­­­­­­­­­­­­­____________
- I live alone: □ yes □ no
- I prepare my medications myself:

□ yes □ no, (nurses or relatives prepare the medications for me)

- Highest level of education (please tick the correct answer):
  - Primary
  - Secondary
  - Tertiary
  - Apprenticeship
  - Technical school
  - University

**Part 2. Medication list.**

- List of all medications you are currently regularly taking. (including the medications, which were not prescribed by your GP):

| Medication (Name/active substance) | Dose | Time of taking | Remark |
| --- | --- | --- | --- |
| *For example: Aspirin* | *100mg* | - 1. *or*   *1x on the morning* |  |
|  |  |  |  |
|  |  |  |  |
|  |  |  |  |
|  |  |  |  |
|  |  |  |  |
|  |  |  |  |
|  |  |  |  |
|  |  |  |  |
|  |  |  |  |
|  |  |  |  |
|  |  |  |  |
|  |  |  |  |
|  |  |  |  |
|  |  |  |  |
|  |  |  |  |

If you need more place, you can write on the back of this piece of paper.

# Part 3. Diagnoses

Please write all of your current diagnoses.

You will find below a list of the most frequent chronic diseases in older adults. If you suffer from one of them, you can tick the box. Including if the disease has been treated successfully. For example hypertension: if you’re currently taking a medication for high blood pressureyou’re your blood pressure is now normal, please mention the disease. Furthermore we ask you to write on the next page all the diseases you are suffering from and which do not figure on the list. Please mention, if you have/had cancer.

| - Hypertension |
| --- |
| - Diabetes Mellitus |
| - Chronic lung disease (COPD, Asthma) |
| - Depression |
| - Coronary heart disease or previous heart infarction (including Angina pectoris, coronary Bypass, coronary stent) |
| - Chronic kidney disease (impaired kidney function, dialysis, kidney transplantation) |
| - Cancer |
| - Psychiatric disease (schizophrenia, bipolar disorder, psychosis, developmental disorder) |
| - Stroke (Stroke, transient ischemic attack (TIA), hemiplegia) |
| - Liver disease (liver cirrhosis, Hepatitis B/C) |
| - Stomach, bowel or pancreas disease (ulcer, gastroeosophageal reflux disease, chronic inflammatory bowel disease, colitis ulcerosa, morbus crohn, pancreatitis) |
| - Disease of the connective tissue (rheumatoid arthritis, vasculitis, systemic lupus erythematosus, systemic sclerosis,… ) |
| - Heart failure |
| - Peripheral artery disease (PAD, amputation of one or more extremity/ies, stent in one or more artery/ies) |
| - Neurological disease (epilepsy, multiple sclerosis, spinal canal stenosis, Parkinson, infantile paralysis (Poliomyelitis), cerebral paresis,…) |
| - Dementia |
| - AIDS |
| - Other diseases, from which you also suffered (please use the rest of the sheet to write them down): |

**Part 4. Questions about deprescribing**

In the following we want to know your opinion about deprescribing. There are no correct or wrong answer to the following questions. All the questions are concerning you, even if you didn’t experience this situation yet. Choose the answer which would correspond to your reaction about it. Please mark your answer as clearly as you can. Example:
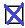


1. Overall, I am satisfied with my current medicines*

| strongly agree  □ | agree  □ | I don’t know  □ | disagree  **□** | strongly disagree  **□** |
| --- | --- | --- | --- | --- |

1. I like to be involved in making decisions about my medicines with my doctors*

| strongly agree  □ | agree  □ | I don’t know  □ | disagree  **□** | strongly disagree  **□** |
| --- | --- | --- | --- | --- |

1. I have a good understanding of the reasons I was prescribed each of my medicines*

| strongly agree  □ | agree  □ | I don’t know  □ | disagree  **□** | strongly disagree  **□** |
| --- | --- | --- | --- | --- |

1. I like to know as much as possible about my medicines*

| strongly agree  □ | agree  □ | I don’t know  □ | disagree  **□** | strongly disagree  **□** |
| --- | --- | --- | --- | --- |

1. I always ask my doctor, pharmacist or other health care professional if there is something I don’t understand about my medicines*

| strongly agree  □ | agree  □ | I don’t know  □ | disagree  **□** | strongly disagree  **□** |
| --- | --- | --- | --- | --- |

1. I know exactly what medicines I am currently taking, and/or I keep an up to date list of my medicines*

| strongly agree  □ | agree  □ | I don’t know  □ | disagree  **□** | strongly disagree  **□** |
| --- | --- | --- | --- | --- |

1. I would be reluctant to stop a medicine that I had been taking for a

long time*

| strongly agree  □ | agree  □ | I don’t know  □ | disagree  **□** | strongly disagree  **□** |
| --- | --- | --- | --- | --- |

1. If one of my medicines was stopped I would be worried about missing out on future benefits.*

| strongly agree  □ | agree  □ | I don’t know  □ | disagree  **□** | strongly disagree  **□** |
| --- | --- | --- | --- | --- |

1. I get stressed whenever changes are made to my medicines.*

| strongly agree  □ | agree  □ | I don’t know  □ | disagree  **□** | strongly disagree  **□** |
| --- | --- | --- | --- | --- |

1. If my doctor recommended stopping a medicine I would feel that he/she was giving up on me*

| strongly agree  □ | agree  □ | I don’t know  □ | disagree  **□** | strongly disagree  **□** |
| --- | --- | --- | --- | --- |

1. have had a bad experience when stopping a medicine before*

| strongly agree  □ | agree  □ | I don’t know  □ | disagree  **□** | strongly disagree  **□** |
| --- | --- | --- | --- | --- |

1. I noticed an improvement due to my medicines.

| strongly agree  □ | agree  □ | I don’t know  □ | disagree  **□** | strongly disagree  **□** |
| --- | --- | --- | --- | --- |

1. B y taking medicines, I feel that I am doing something to help my diseases.

| strongly agree  □ | agree  □ | I don’t know  □ | disagree  **□** | strongly disagree  **□** |
| --- | --- | --- | --- | --- |

1. I feel my GP lacks time and support that would be necessary to discuss stopping one or more medicines.

| strongly agree  □ | agree  □ | I don’t know  □ | disagree  **□** | strongly disagree  **□** |
| --- | --- | --- | --- | --- |

1. I would be reluctant to stop a medicine that was prescribed by a specialist and not by my GP.

| strongly agree  □ | agree  □ | I don’t know  □ | disagree  **□** | strongly disagree  **□** |
| --- | --- | --- | --- | --- |

1. If my GP would tell me I am safe to deprescribe one or more medicines, I still would feel unsure about how to stop them (e.g. stop at once or lower the dosage).

| strongly agree  □ | agree  □ | I don’t know  □ | disagree  **□** | strongly disagree  **□** |
| --- | --- | --- | --- | --- |

1. It happened to me that I did not know how to cease medicines (even after discussing deprescribing with my GP).

| strongly agree  □ | agree  □ | I don’t know  □ | disagree  **□** | strongly disagree  **□** |
| --- | --- | --- | --- | --- |

1. I sometimes feel I have to take my medicines to ‘please’ my family.

| strongly agree  □ | agree  □ | I don’t know  □ | disagree  **□** | strongly disagree  **□** |
| --- | --- | --- | --- | --- |

1. I am old so why change my medication?

| strongly agree  □ | agree  □ | I don’t know  □ | disagree  **□** | strongly disagree  **□** |
| --- | --- | --- | --- | --- |

1. Do you think there are other reasons why you wouldn’t reduce or stop medications? (Please describe these reasons below)

__________________________________________________________________________________________________________________

1. If my doctor said it was possible I would be willing to stop one or more of my regular medicines.*

| strongly agree  □ | agree  □ | I don’t know  □ | disagree  **□** | strongly disagree  **□** |
| --- | --- | --- | --- | --- |

1. I spend a lot of money on my medicines*

| strongly agree  □ | agree  □ | I don’t know  □ | disagree  **□** | strongly disagree  **□** |
| --- | --- | --- | --- | --- |

1. Taking my medicines every day is very inconvenient.*

| strongly agree  □ | agree  □ | I don’t know  □ | disagree  **□** | strongly disagree  **□** |
| --- | --- | --- | --- | --- |

1. I feel that I am taking a large number of medicines*

| strongly agree  □ | agree  □ | I don’t know  □ | disagree  **□** | strongly disagree  **□** |
| --- | --- | --- | --- | --- |

1. I feel that my medicines are a burden to me*

| strongly agree  □ | agree  □ | I don’t know  □ | disagree  **□** | strongly disagree  **□** |
| --- | --- | --- | --- | --- |

1. Sometimes I think I take too many medicines*

| strongly agree  □ | agree  □ | I don’t know  □ | disagree  **□** | strongly disagree  **□** |
| --- | --- | --- | --- | --- |

1. I feel that I may be taking one or more medicines that I no longer need*

| strongly agree  □ | agree  □ | I don’t know  □ | disagree  **□** | strongly disagree  **□** |
| --- | --- | --- | --- | --- |

1. I would like to try stopping one of my medicines to see how I feel without it*

| strongly agree  □ | agree  □ | I don’t know  □ | disagree  **□** | strongly disagree  **□** |
| --- | --- | --- | --- | --- |

1. I would like my doctor to reduce the dose of one or more of my medicines.*

| strongly agree  □ | agree  □ | I don’t know  □ | disagree  **□** | strongly disagree  **□** |
| --- | --- | --- | --- | --- |

1. I think one or more of my medicines may not be working*

| strongly agree  □ | agree  □ | I don’t know  □ | disagree  **□** | strongly disagree  **□** |
| --- | --- | --- | --- | --- |

1. I believe one or more of my medicines may be currently giving me side effects*

| strongly agree  □ | agree  □ | I don’t know  □ | disagree  **□** | strongly disagree  **□** |
| --- | --- | --- | --- | --- |

1. I have a good relationship with my GP that would make me feel comfortable and safe regarding deprescribing.

| strongly agree  □ | agree  □ | I don’t know  □ | disagree  **□** | strongly disagree  **□** |
| --- | --- | --- | --- | --- |

1. If new studies would tell me there is an avoidable risk of too much medicines, that would make me want to deprescribe.

| strongly agree  □ | agree  □ | I don’t know  □ | disagree  **□** | strongly disagree  **□** |
| --- | --- | --- | --- | --- |

1. I generally don’t like to take medicines.

| strongly agree  □ | agree  □ | I don’t know  □ | disagree  **□** | strongly disagree  **□** |
| --- | --- | --- | --- | --- |

1. Stopping one or more medicines would make me feel getting back control in my life.

| strongly agree  □ | agree  □ | I don’t know  □ | disagree  **□** | strongly disagree  **□** |
| --- | --- | --- | --- | --- |

Do you think there are other reasons why you would like to reduce or stop medications? (Please describe these reasons below)

_______________________________________________________________

_______________________________________________________________

_______________________________________________________________

_______________________________________________________________

_______________________________________________________________

**Your comments are very welcome:**

___________________________________________________________________

___________________________________________________________________

___________________________________________________________________

___________________________________________________________________

___________________________________________________________________

**The end**

Thank you very much for participating in our study.

We appreciate the fact that you took time for our questionnaire. Please return the questionnaire to your GP’s office.
